# Impact of integrated care coordination on pediatric asthma hospital presentations

**DOI:** 10.3389/fped.2022.929819

**Published:** 2022-09-23

**Authors:** Nusrat Homaira, Emma Dickins, Stephanie Hodgson, Mei Chan, Sandra Wales, Melinda Gray, Sarah Donnelly, Christine Burns, Louisa Owens, Michael Plaister, Anthony Flynn, Jennifer Andresen, Kimberley Keane, Karen Wheeler, Bronwyn Gould, Nadine Shaw, Adam Jaffe, Christie Breen, Lisa Altman, Susan Woolfenden

**Affiliations:** ^1^Discipline of Paediatrics and Child Health, Faculty of Medicine and Health, UNSW Sydney, Kensington, NSW, Australia; ^2^Sydney Children's Hospital, Sydney, NSW, Australia; ^3^Integrated Care, Sydney Children's Hospital Network, Sydney, NSW, Australia; ^4^Asthma Australia, Melbourne, VIC, Australia; ^5^Rozelle Medical Centre, Sydney, NSW, Australia; ^6^Central and Eastern Sydney Public Health Network, Sydney, NSW, Australia; ^7^General Practitioner, Sydney, NSW, Australia

**Keywords:** childhood asthma, care coordination, integrated care, healthcare utilization, model of care

## Abstract

**Introduction:**

Frequent asthma attacks in children result in unscheduled hospital presentations. Patient centered care coordination can reduce asthma hospital presentations. In 2016, The Sydney Children's Hospitals Network launched the Asthma Follow up Integrated Care Initiative with the aim to reduce pediatric asthma emergency department (ED) presentations by 50% through developing and testing an integrated model of care led by care coordinators (CCs).

**Methods:**

The integrated model of care was developed by a multidisciplinary team at Sydney Children's Hospital Randwick (SCH,R) and implemented in two phases: Phase I and Phase II. Children aged 2–16 years who presented ≥4 times to the ED of the SCH,R in the preceding 12 months were enrolled in Phase I and those who had ≥4 ED presentations and ≥1 hospital admissions with asthma attack were enrolled in Phase II. Phase I included a suite of interventions delivered by CCs including encouraging parents/carers to schedule follow-up visits with GP post-discharge, ensuring parents/carers are provided with standard asthma resource pack, offering referrals to asthma education sessions, sending a letter to the child's GP advising of the child's recent hospital presentation and coordinating asthma education webinar for GPs. In addition, in Phase II CCs sent text messages to parents/carers reminding them to follow-up with the child's GP. We compared the change in ED visits and hospital admissions at baseline (6 months pre-enrolment) and at 6-and 12-months post-enrolment in the program.

**Results:**

During December 2016-January 2021, 160 children (99 in Phase I and 61 in Phase II) were enrolled. Compared to baseline at 6- and 12-months post-enrolment, the proportion of children requiring ≥1 asthma ED presentations reduced by 43 and 61% in Phase I and 41 and 66% in Phase II. Similarly, the proportion of children requiring ≥1 asthma hospital admissions at 6- and 12-months post-enrolment reduced by 40 and 47% in Phase I and 62 and 69% in Phase II.

**Conclusion:**

Our results support that care coordinator led integrated model of asthma care which enables integration of acute and primary care services and provides families with asthma resources and education can reduce asthma hospital presentations in children.

## Introduction

Asthma is the most common chronic respiratory condition of childhood affecting more than 14% of the children globally (1). In Australia, about 10% of children aged < 14 years are affected by the condition (2). In 2015, asthma was the leading cause of disease burden in Australian children aged 5–14 years (2). In New South Wales (NSW), the most densely populated state of the country, there are >10,000 children presenting to emergency department (ED) and 3,000 children hospitalized with acute asthma attacks every year (3). Frequent hospital presentations with an asthma attack are a marker of poorly controlled disease. The reasons for poor progress in pediatric asthma control are multifaceted, including inadequate asthma education, failure to mitigate environmental triggers, lack of coordination within and between healthcare services and sub-optimal support in the community (4). Because of the potential opportunity to address these challenges in the community, i.e. outside the acute care environment, there is growing interest in care coordination as an effective strategy to reduce burden asthma health utilization. Care coordination is a patient-centered multidisciplinary approach integrating health care and social support to develop a comprehensive model of care whereby evidence-based care is monitored by care coordinators (CCs) (5). CCs can support families navigate through the complex health system and reduce hospital presentations in patients with chronic illnesses (6). Evidence based post-discharge care coordination can reduce asthma hospitalization by 80% (7). Over the years, numerous asthma optimisation programs have been developed in Australia in different care settings (8). However, the role of care coordination and service integration has not been explored.

In 2016, we reviewed 30 children who presented multiple times (≥ 4 times) with an asthma attack and/or wheezing episodes to the ED of the Sydney Children's Hospital, Randwick (SCH,R). We found that these ‘frequent presenters' presented to the ED 286 times in a 12-month period of which 214 times were due to an asthma attack. Feedback from parents during the review showed there was anxiety about how to manage asthma, which asthma action plan to use, and a misconception that their child's asthma should be managed by SCH, R(tertiary institute) instead of their General Practitioners (GP). Our findings corroborated with other studies where parents /carers of children requiring hospital presentations for asthma attacks reported not having asthma action plans, not attending follow-up visits with primary care providers and using ED as the first point of care (9). These findings highlighted that children who present frequently to the ED with asthma attacks and are then discharged, represent a group who do not have optimal support and care in the community. Therefore, in 2016 The Sydney Children's Hospitals Network (SCHN), launched the Asthma Follow Up Integrated Care Initiative. The aim of the initiative was to reduce pediatric asthma ED re-presentations by 50% through developing and testing an integrated coordinated model of asthma care. In this report we present the impact of the Asthma Follow Up Integrated Care Initiative on pediatric asthma hospital presentation.

## Methods

### Study design and setting

In 2021, we conducted a pre- and post-implementation cohort evaluation of the Asthma Follow Up Integrated Care Initiative implemented at SCH, R. The SCH, R is one of two hospitals within the SCHN. The SCHN is the leading tertiary pediatric care provider in Australia and provides approximately 90% of acute care for children in NSW.

### Participants

Children aged 2–16 years and their families who presented ≥ 4 times to the ED of the SCH,R of which three or more were due to asthma attacks in the preceding 12 months, were identified as a group who are at risk of presenting frequently to the hospital with an asthma attack (10), and were eligible to be enrolled in the Asthma Follow Up Integrated Care Initiative. Children who required admission in to the intensive care unit for an asthma attack, or were under the care of respiratory department at SCH, R were excluded.

### Intervention

The SCHN Asthma Follow Up Integrated Care Initiative assembled a multidisciplinary integrated care team consisting of general pediatrician, respiratory pediatricians, emergency department nurse specialist, asthma clinical nurse consultant, GPs, pediatric care coordinators, researchers, health service managers and parents of children to develop the model of care. Through a series of iterative consultations, the team identified change ideas including improving identification of ‘frequent presenters', ensuring follow-up with primary care providers after discharge from hospital, enhancing access to primary care providers, decreasing variability in asthma management and improving parental understanding of asthma management. Based on the review of the evidence (11–13) the team came to the consensus that care coordination to deliver an integrated model of care addressing the identified issues is a feasible program. Thus, an integrated model of care led by CCs was implemented within the SCH, R. The CCs were part of the larger integrated care program of SCH,R and were involved in coordinating care for children with medical complexities and who visited the hospital frequently (14).

The Asthma Follow Up Integrated Care Initiative had two phases: Phase I and Phase II. Phase I commenced in December 2016 and included children aged 2–16 years and their families who presented to the ED of the SCHN, *R* ≥ 4 times, including three or more of those presentations due to asthma attack, in the preceding 12 months. Phase II began in July 2018 which included similar care coordination for children (aged 2–16 years) who presented to the ED ≥ 4 times with asthma attack in the preceding 12 months ED including three or more of those presentations due to asthma attack and additionally had to have one or more asthma hospital admissions in the preceding 12 months. Phase 2 was initiated to capture children who may have greater need of care coordination but were not under the care of respiratory department.

A flagging system was developed in the hospital electronic medical records system of SCH,R which was used to notify CCs of eligible children. The CCs invited parents/carers of eligible children to participate in the integrated care initiative. The integrated model of care included a suite of interventions delivered by the CC:

- Verbally encouraging parents/carers upon discharge to schedule a follow-up visit with the child's GP within 2–3 days post-discharge.
- Ensuring parents/carers were provided with a standardized asthma resource pack upon discharge from ED. The resource pack included individualized Asthma Action Plans and discharge instructions, Aiming for Asthma Improvement in Children (AAIC) developed asthma booklet ‘Asthma and Your Child' developed by the Aiming for Asthma Improvement in Children (AAIC) program of SCH,R and information about free upcoming asthma education sessions organized by AAIC of SCH,R The asthma resource pack was printed by the CC and made available in the ED to be provided by ED staff to eligible children at the time of discharge.
- Sending a letter to the child's GP advising of the child's recent hospital presentation. This letter contained pediatric asthma management best practice points and also included recommendation for influenza vaccination for the child, review of child's asthma action plan, and understanding preventer medication requirements and medication delivery device technique, and referral to a pediatrician if necessary.
- Coordinating an asthma education webinar for GPs in collaboration with respiratory pediatricians and asthma nurses within SCH,R. The webinar was promoted through the Central and Eastern Sydney Primary Health Network to all GPs in the surrounding area. A total of 15 GPs attended the webinar and a recording was made available for further access.
- Additionally, to ensure continuity of care in Phase II the CCs sent a reminder text message to parents of eligible children within 2–3 days post-discharge from hospital reminding them to follow-up with the child's GP, review their child's asthma action plan, encourage attendance to free asthma education sessions and take the child for influenza vaccination.

### Outcome measures

We determined the change in health service utilization (ED visits and hospital admissions) at baseline (6 months pre-enrolment) and at 6-and 12-months after enrolment in the program (15). These data were extracted retrospectively from the electronic medical records database, with the assistance of the Patient Information Unit at the SCH,R. Children enrolled in the program with completed post-enrolment records of hospital presentations for ≥ 12 months were included in the analysis.

### Sample size

The SCHN Asthma Follow Up Integrated Care Initiative was a quality improvement project, and all eligible children were included for care coordination. This study evaluated the Asthma Follow Up Integrated Care Initiative and no specific sample size calculation was done.

### Statistical analysis

Descriptive statistics including mean, median, standard deviation (SD), range, interquartile range (IQR) and percentage were reported, where appropriate. Comparisons of background characteristics between Phase I and Phase II were analyzed using Chi-squared test for binomial data and student's *t* test for continuous data.

The repeated measurements of ED visits and hospital admissions in the preceding 6 months at baseline (pre-enrolment), 6-months and 12-months post-enrolment were analyzed using generalized estimating equation (GEE) assuming Poisson distribution and exchangeable correlation structure (16). All models were adjusted for sex, age, Socio-Economic Indexes of Areas (SEIFA) and country of birth. Socio-economic disadvantage was derived from the postcode of residence based on the decile ranking using the SEIFA and Index of Relative Socioeconomic Advantage and Disadvantage (IRSAD) compiled by the Australian Bureau of Statistic (17). The decile ranking means that areas are divided up into ten equal sized groups, depending on their score. All areas based on their postcodes are ordered from lowest to highest score, then the lowest 10% of areas are given a decile number of 1, the next lowest 10% of areas are given a decile number 2 and so on, up to the highest 10% of areas which are given a decile number of 10. The estimated rate ratios (RRs) with 95% confidence intervals (CIs) of ED visits and hospital admissions at 6-month and 12-month post-enrolment were calculated in comparison to that of the 6 months pre-intervention measurements obtained at baseline. Subgroup analyses were performed by stratifying the cohort into Phase I and Phase II to assess the differences in hospital utilizations for the two group. All statistical analyses were conducted using R program 4.1.0 version with 5% level of significance.

As lockdowns and other public health measures implemented to reduce the transmission of the COVID-19 pandemic led to reduction in hospital presentation due to chronic conditions in 2020 (18), we also analyzed the data excluding the pandemic year (2020) (Supplementary material).

### Ethics approval

All study participants provided informed consent to be enrolled in the care initiative program. This study was approved by the Sydney Children's Hospitals Network Human Ethics Committee (LNR/15/SCHN/299).

## Results

### Background characteristics

During December 2016-January 2021, 160 children including 99 in Phase I and 61 in Phase II were included in the Asthma Follow Up Integrated Care Initiative. As shown in Table 1, the majority of the children were male, median age 3 years (IQR = 2–4 years). Most of them (96%) were born in Australia with about 54% lived in areas of SEIFA decile ranking≥ 8. There were no significant differences in the background characteristics of children in Phase I and Phase II.

**Table 1:** Background characteristics of children enrolled in the asthma follow-up integrated care initiative (December 2016- January 2021).

	**Total**		**Phase I**		**Phase II**		***p*** **-value**
**n**	200		99		101		
**Gender**							0.0012^*^
Female (%)	59	(30%)	23	(23%)	36	(36%)	
Male (%)	141	(70%)	76	(77%)	65	(64%)	
**Age (year)**							0.95^**^
Mean (SD)	3.39	(2.42)	3.38	(2.53)	3.40	(2.31)	
Median (IQR)	3	(2–4)	3	(2–4)	3	(2–5)	
Range	1–15		1–15		1–12		
**SEIFA**							0.13^*^
Decile ranking 5	11	(6%)	5	(5%)	6	(6%)	
Decile ranking 6	17	(9%)	11	(11%)	6	(6%)	
Decile ranking 7	46	(23%)	20	(20%)	26	(26%)	
Decile ranking 8	38	(19%)	19	(19%)	19	(19%)	
Decile ranking 9	65	(33%)	34	(34%)	31	(31%)	
Decile ranking 10	23	(12%)	10	(10%)	13	(13%)	
**Country of birth**							0.28^*^
Australia	192	(96%)	94	(95%)	98	(97%)	
Overseas	8	(4%)	5	(5%)	3	(3%)	
**Aboriginal**							0.24^*^
Yes	1	(0.5%)	1	(1%)	0	(0%)	
No	199	(99.5%)	98	(99%)	101	(100%)	

* p-value for chi-squared test;

** p-value for student's t test.

SD, standard deviation; IQR, Interquartile range; SEIFA, Socio-Economic Indexes for Areas.

### Emergency department visits

**Figure 1:**
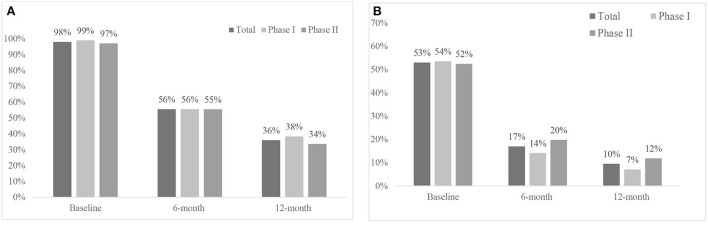
Change in hospital utilization in the preceding 6 months at baseline, 6months, and 12months after implementation of asthma follow-up integrated care initiative, Sydney Children's Hospital, December 2016-January 2021. **(A)** Proportion of children with ≥1ED visits: Phase I: 43% reduction at 6 months and 61% at 12 months (*p* < 0.001). Phase II: 42% reduction at 6 months and 63% at 12 months (*p* < 0.001). **(B)** Proportion of children with ≥1 hospital admission: Phase I: 40% reduction at 6 months and 34% at 12 months (*p* < 0.001). Phase II: 47% reduction at 6 months and 42% at 12 months (*p* < 0.001).

Overall, 10,058 (99%) children had attended ED for asthma in the 6 months period before they were enrolled in the program. The proportion of children requiring ≥1 ED presentations for asthma reduced by 42% at 6-month and 62% by 12-month (*p* < 0.001) during the post-enrolment period compared to baseline (Figure 1A). The frequency of ED visits over 6 months period also declined after the implementation of the program; from a median of 3 at baseline to 1 at 6 month and 0 at 12 months for Phase I and Phase II (Table 2). The risk of ED visits over a six-month period was reduced by 67% at 6-month and 80% at 12-month time during the post-enrolment period (Table 3). Similar results were found in both Phase I and Phase II cohorts (Table 3).

**Table 2:** Frequency of asthma emergency department visits and hospital admissions in preceding 6 moths at abseline and at 6-months and 12-months post intervention, Sydney Children's Hospital, December 2016-January 2021.

	**Phase I**			**Phase II**		
	**Baseline**	**6-month time**	**12-month time**	**Baseline**	**6-month time**	**12-month time**
**Asthma emergency department visits (n)**						
Mean (SD)	3.1 (1.5)	1.0 (1.3)	0.6 (1)	2.9 (1.4)	1 (1.3)	0.6 (1.1)
Median (IQR)	3 (2–4)	1 (0–1.5)	0 (0–1)	3 (2–4)	1 (0–1)	0 (0–1)
Range	0–10	0–6	0–5	0–8	0–7	0–7
**Asthma hospital admissions (n)**						
Mean (SD)	0.8 (0.9)	0.14 (0.4)	0.07 (0.3)	0.9 (1.1)	0.3(0.5)	0.2 (0.5)
Median (IQR)	1(0–1)	0 (0–0)	0 (0–0)	1(0–1)	0 (0–0)	0 (0–0)
Range	0–4	0–2	0–3	0–4	0–1	0–1

ED, Emergency department; SD, standard deviation; IQR, Interquartile range.

**Table 3:** Risk of hospital utilization before and after implementation of asthma follow-up integrated care initiative, Sydney Children's Hospital, December 2016-January 2021.

	**Pre-implementation**	**6-month post-implementation**		**12-month post-implementation**	
	**Baseline**	**RR (95% CI)**	***p*** **-value**	**RR (95% CI)**	***p*** **-value**
**Total**					
ED visits	Ref	0.34 (0.28–0.40)	< 0.001	0.20 (0.16–0.26)	< 0.001
Hospital admissions	Ref	0.24 (0.17–0.34)	< 0.001	0.13 (0.08–0.22)	< 0.001
**Phase I**					
ED visits	Ref	0.33 (0.26–0.42)	< 0.001	0.20 (0.14–0.27)	< 0.001
Hospital admissions	Ref	0.19 (0.11–0.32)	< 0.001	0.09 (0.05–0.19)	< 0.001
**Phase II**					
ED visits	Ref	0.34 (0.27–0.43)	< 0.001	0.21 (0.14–0.30)	< 0.001
Hospital admissions	Ref	0.28 (0.18–0.44)	< 0.001	0.17 (0.09–0.31)	< 0.001

RR, Rate ratio as determined by generalized estimating equation regression; 95% CI, 95% confidence interval; ED, Emergency department.

All models were adjusted for sex, age, SEIFA and country of birth.

### Hospital admissions

In total 106 (66%) children had ≥1 asthma hospital admissions in the 6 months period before they were enrolled in the program The proportion of children who required ≥1 hospital admissions for asthma were reduced by 48 and 55% in the 6-month and 12-month (*p* < 0.001) post-enrolment period respectively compared to proportion of children requiring hospital admissions at baseline (Figure 1B). The median number of hospital admissions went from one at baseline to 0 at 6-month and 12-month post intervention (Table 2). When comparing the pre- and post-enrolment periods, there risk of hospital admission decreased significantly in both Phase I (81–91%) and Phase II (79–84%) compared to baseline (Table 3).

## Discussion

Our findings support that a care coordinator led integrated model of asthma care which enables integration of acute and primary care services and provides families with asthma resources and education can reduce asthma hospital presentation in children (19). In addition, the asthma coordinated model of care was able to achieve the target (50% reduction in ED presentations) set out at the beginning of the program. The observed reduction in asthma hospital presentation for children in both Phase I and II may have been due to person-centered care coordination which enabled linkage between parents/carers and primary care providers ensuring follow-up visits with primary care providers and reducing the use of ED as the primary point of care (20). In addition to organizing referral letters for follow-up visits in Phase I, Phase II also included text message reminders for follow-up with GPs. The Australian Asthma Handbook recommends that children have a follow-up visit within three days post a discharge from hospital due to an asthma attack (21). Follow-up visits with primary care providers post hospital presentation with an asthma attack is imperative to review patient's asthma action plan, medication adherence, inhaler device use skills and environmental triggers and has been linked with improved health outcomes including reduced need for repeat hospital presentation in children with asthma (22). Although we could not ascertain how many parents/carers took their children for follow-up visits with their GPs post-discharge from the hospital, the sustained reduction in hospital presentation at 12 moths post-implementation suggest that pro-active care coordination that facilitated referral services may have led to the reduction in hospital utilization (23). Follow-up visits with primary care providers can increase by 20% when patients receive appointment assistance compared to usual care (20). Sharing of asthma information between acute care and primary care providers may have also contributed to the observed reduction in asthma-related hospital presentations. Indeed programs that provide decision support tools to primary care providers have been shown to reduce asthma morbidity including ED visits and hospitalizations (24, 25).

We observed a 40% reduction in asthma ED presentations at 6 months post intervention compared to baseline for children in Phases I and II which was maintained at just over 60% at 12-months. This reduction was comparable to a community asthma initiative implemented in the US (26). However, the model of care in the US was different from our care initiative. In addition to care coordination, the US model of care included home visits to assess home environmental triggers. Indoor housing conditions and exposure to allergens such as inadequate ventilation, environmental tobacco smoke, pests etc. have been linked to asthma attacks (27, 28). Incorporating home environment assessment as part of care coordination can identify important modifiable factors associated in children with problematic asthma (28).Due to logistical constraints, we were not able to provide home visits which may explain the greater reduction in hospital admissions (84.8%) observed in the US study compared to our findings (~40%). As the integrated asthma care initiative evolves it will be important to revisit the role of care coordinator and optimize the suite of interventions.

The suite of interventions included in integrated models of care vary between different settings (29). While our integrated model of asthma care had five interventions including enhanced linkages between primary and acute care services, improving asthma follow-up visits and asthma education for parents and primary care providers, the number of interventions included in different integrated models of care can vary between one to nine (29). However, like our model of care, the majority of the integrated models of care are facilitated by a care coordinator highlighting the need for personalized coordinated care to improve health outcomes for children with chronic conditions such as asthma (29). Development of a standard model of integrated asthma care comprising essential interventions to improve health outcomes in children will help in translating the model and comparing the impact of integrated asthma care across different settings.

One of the major limitations of this study is it was a quasi-experimental design without a comparison group. However, a similar approach has been used in evaluating other care coordination models for children in different settings (30, 31). We could not rule out the possibility that the decreasing pattern in healthcare utilization may have been due to natural progression of the disease as there was no control group. Nevertheless, our findings corroborate with other studies with control children suggesting the pattern observed was potentially due to care coordination (32). Although our care initiative was implemented only within one hospital, SCH, R is one of the major pediatric tertiary hospitals in Australia and caters to a diverse range of children from varying sociodemographic background. The Asthma Follow Up Integrated Care was part of larger integrated care program within SCH,R which showed that the direct out-of-pocket and productivity cost savings to families due to care coordination were estimated at AU$146,661 (33). However, we did not evaluate cost-effectiveness of the specific asthma intervention which is an important component for policy decision around wider roll out of models of care. In addition, even though care coordination is increasingly being recognized as an effective way to reduce the burden health care utilization, the cost associated with care coordination maybe prohibitive in low middle income countries (LMICs). Although service cost for integrated care initiative remains unclear (34), future randomized controlled trials incorporating cost analysis will help validate our findings and sustainability of the model of care.

Following successful outcome, the Asthma Follow Up Integrated Care Initiative has now been incorporated as standard clinical care within SCH, R. The multidisciplinary approach in developing and implementing the integrated asthma care model ensured engagement of relevant stakeholders and adoption of the model as routine care. A systematic approach to ongoing evaluation of the care coordination approach in managing children who frequently present to the hospital with asthma will help in adaptation and refinement of the model of care. Further research is needed to discern the impact of care coordination on a child's asthma-related quality of life and parents/carers quality of life and on the direct and indirect cost associated with frequent asthma hospital presentations.

## Data availability statement

The data analyzed in this study is subject to the following licenses/restrictions. Access to the dataset analyzed during the current study is not permitted without the express permission of the approving human research ethics committees and data custodians. There is no additional data available. Requests to access these datasets should be directed to Sydney Children's Hospital https://www.schn.health.nsw.gov.au/hospitals/sch/.

## Ethics statement

The studies involving human participants were reviewed and approved by the Sydney Children's Hospitals Network Human Ethics Committee. Written informed consent to participate in this study was provided by the participants' legal guardian/next of kin. Written informed consent was obtained from the minor(s)' legal guardian/next of kin for the publication of any potentially identifiable images or data included in this article.

## Author contributions

NH, SWa, MG, CBu, LO, MP, AF, NS, AJ, CBr, LA, and SWo conceived and designed the study. ED, SH, SD, JA, KK, KW, and BG implemented the study. MC performed the statistical analyses for the study. NH was responsible for drafting the manuscript. All authors provided critical feedback with drafting of the manuscript. All authors contributed to the article and approved the submitted version.

## Funding

The project manager (LA) and clinical lead (SWo) were funded through the NSW Ministry of Health's Innovator Funding for Integrated Care. NH was funded through Research Fellowship of the National Health and Medical research Council of the Australian Government (GNT1158646).

## Conflict of interest

The authors declare that the research was conducted in the absence of any commercial or financial relationships that could be construed as a potential conflict of interest.

## Publisher's note

All claims expressed in this article are solely those of the authors and do not necessarily represent those of their affiliated organizations, or those of the publisher, the editors and the reviewers. Any product that may be evaluated in this article, or claim that may be made by its manufacturer, is not guaranteed or endorsed by the publisher.
