# Supplementary material for: Impact of integrated care coordination on pediatric asthma hospital presentations
Source: Front Pediatr. 2022 Sep 23;10:929819. doi: 10.3389/fped.2022.929819 (PMC9537948; doi:10.3389/fped.2022.929819)
Supplement: Supplementary file 1 [file Data_Sheet_1.pdf]

Supplementary Table 1. Background characteristics of children enrolled in the asthma follow-up integrated care initiative (2016-2019)

	Total		Phase I		Phase II		*p-value*
**n**	127		82		45		
**Gender**							0.15*
Female (%)	34	(27%)	18	(22%)	16	(36%)	
Male (%)	93	(73%)	64	(78%)	29	(64%)	
**Age (year)**							
mean (SD)	3.21	(2.36)	3.37	(2.52)	2.93	(2.02)	0.30**
Median (IQR)	3	(2-4)	3	(2-4)	2	(2-4)	
Range	1-15		1-15		1-12		
**SEIFA**							0.97*
Decile ranking 5	7	(6%)	5	(6%)	2	(4%)	
Decile ranking 6	11	(9%)	7	(9%)	4	(9%)	
Decile ranking 7	26	(20%)	16	(20%)	10	(22%)	
Decile ranking 8	25	(20%)	15	(18%)	10	(22%)	
Decile ranking 9	45	(35%)	31	(38%)	14	(31%)	
Decile ranking 10	13	(10%)	8	(10%)	5	(11%)	
**Country of Birth**							1.00*
Australia	121	(95%)	78	(95%)	43	(96%)	
Oversea	6	(5%)	4	(5%)	2	(4%)	
**Aboriginal**							1.00*
Yes	1	(1%)	1	(1%)	0	(0%)	
No	126	(99%)	81	(99%)	45	(100%)	

*p-value for chi-squared test; **p-value for student's *t* test

SD=standard deviation; IQR=Interquartile range; SEIFA=Socio-Economic Indexes for Areas

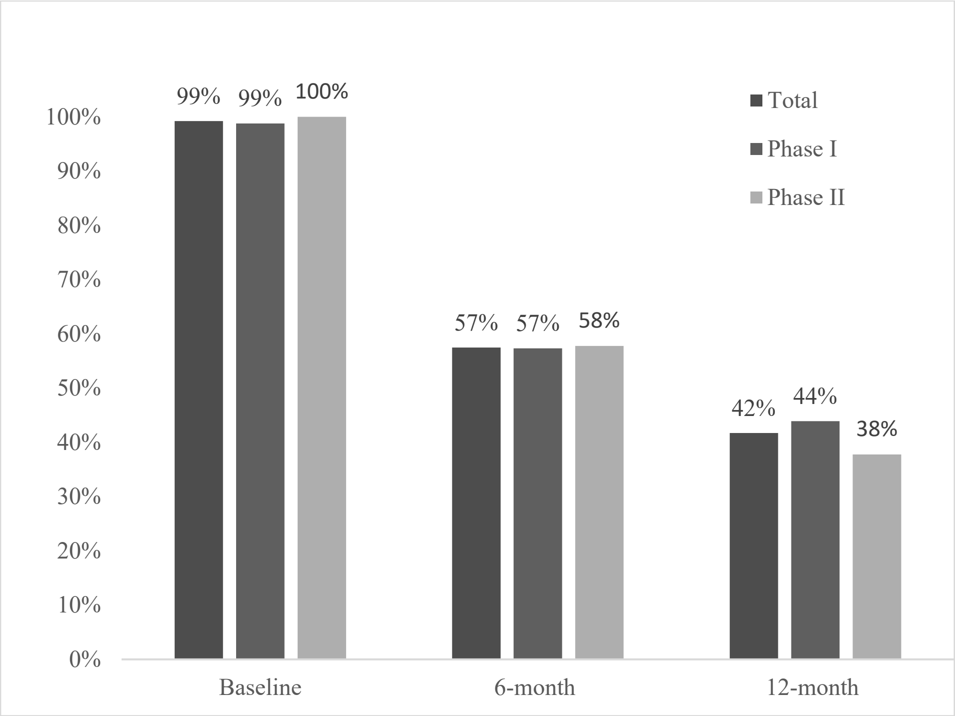

Figure 1a. Proportion of children with ≥ 1 ED visit:
Phase I 42% reduction at 6 months and 55% at 12 months (*p* < 0.001)
Phase II: 42% reduction at 6 months and 62% at 12 months (*p* <0.001)

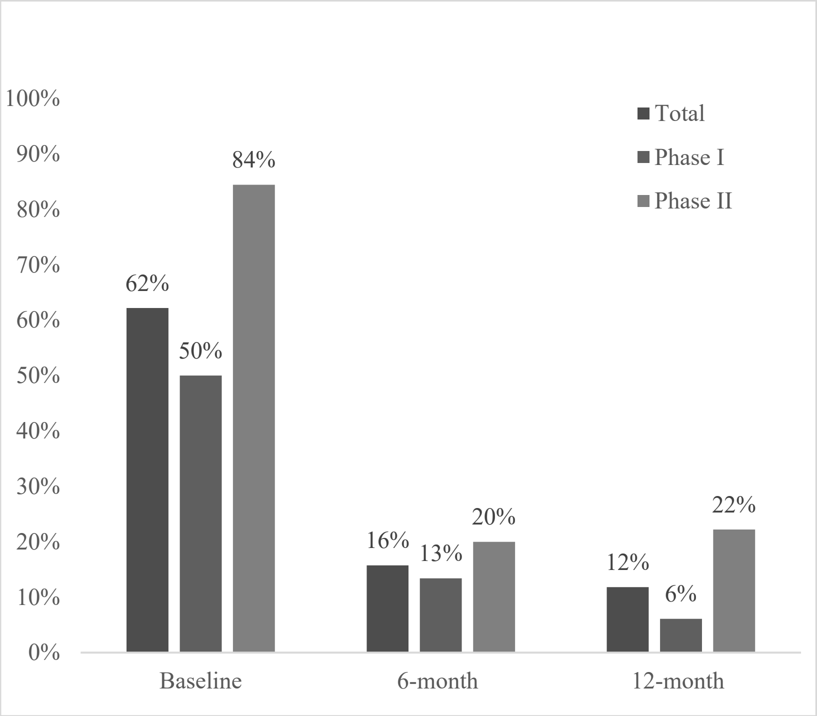

Figure 1b. Proportion of children with ≥ 1 hospital admission:
Phase I 37% reduction at 6 months and 44% at 12 months (*p* < 0.001)
Phase II: 64% reduction at 6 months and 62% at 12 months (*p* < 0.001)

Supplementary Figure 1. Change in hospital utilisation in the preceding 6 months at baseline, 6 months and 12 months after implementation of asthma follow-up integrated care initiative, Sydney Children's Hospital, 2016-2019

Supplementary Table 2. Frequency of asthma emergency department visits and hospital admissions in preceding 6 months at baseline and at 6-months and 12-months post-intervention, Sydney Children's Hospital 2016-2019

	**Phase I**			**Phase II**		
	**Baseline**	**6-month time**	**12-month time**	**Baseline**	**6-month time**	**12-month time**
**Asthma emergency department visits (n)**						
Mean (SD)	3.0 (1.5)	1.1 (1.4)	0.7 (1.0)	3.0 (1.3)	1.0 (1.1)	0.7 (1.1)
Median (IQR)	3 (2-4)	1 (0-2)	0 (0-1)	3 (2-4)	1 (0-2)	0 (0-1)
Range	0-10	0-6	0-5	1-6	0-4	0-4
**Asthma hospital admissions (n)**						
Mean (SD)	0.7 (0.9)	0.1 (0.3)	0.1 (0.2)	1.5 (1.0)	0.3 (0.6)	0.2 (0.5)
Median (IQR)	0.5 (0-1)	0 (0-0)	0 (0-0)	1 (1-2)	0 (0-0)	0 (0-0)
Range	0-4	0-1	0-1	0-4	0-2	0-2

SD=standard deviation; IQR=Interquartile range

Supplementary Table 3. Comparisons of hospital utilisation before and after implementation of asthma follow-up integrated care initiative, Sydney Children's Hospital 2016-2019

	Pre-implementation	6-month post-implementation		12-month post-implementation	
	**Baseline**	**RR (95% CI)**	***p-value***	**RR (95% CI)**	***p-value***
**Total**					
ED visit	ref	0.36 (0.29-0.43)	< 0.001	0.22 (0.17-0.29)	< 0.001
Hospital admission	ref	0.19 (0.12-0.30)	< 0.001	0.13 (0.08-0.21)	< 0.001
**Phase I**					
ED visit	ref	0.37 (0.27-0.48)	< 0.001	0.23 (0.16-0.31)	< 0.001
Hospital admission	ref	0.19 (0.10-0.35)	< 0.001	0.08 (0.04-0.20)	< 0.001
**Phase II**					
ED visit	ref	0.33 (0.24-0.45)	< 0.001	0.24 (0.15-0.37)	< 0.001
Hospital admission	ref	0.20 (0.10-0.38)	< 0.001	0.17 (0.09-0.31)	< 0.001

RR=Rate ratio as determined by generalised estimating equation regression; 95% CI=95% confidence interval; ED=Emergency department

Note: all models were adjusted for sex age, SEIFA and country of birth
